# Hepatocyte apical bulkheads provide a mechanical means to oppose bile pressure

**DOI:** 10.1083/jcb.202208002

**Published:** 2023-01-30

**Authors:** Maarten P. Bebelman, Matthew J. Bovyn, Carlotta M. Mayer, Julien Delpierre, Ronald Naumann, Nuno P. Martins, Alf Honigmann, Yannis Kalaidzidis, Pierre A. Haas, Marino Zerial

**Affiliations:** 1 Max Planck Institute of Molecular Cell Biology and Genetics, Dresden, Germany; 2 Center for Systems Biology Dresden, Dresden, Germany; 3 Max Planck Institute for the Physics of Complex Systems, Dresden, Germany; 4 Cluster of Excellence Physics of Life, Technische Universität Dresden, Dresden, Germany

## Abstract

Hepatocytes grow their apical surfaces anisotropically to generate a 3D network of bile canaliculi (BC). BC elongation is ensured by apical bulkheads, membrane extensions that traverse the lumen and connect juxtaposed hepatocytes. We hypothesize that apical bulkheads are mechanical elements that shape the BC lumen in liver development but also counteract elevated biliary pressure. Here, by resolving their structure using STED microscopy, we found that they are sealed by tight junction loops, connected by adherens junctions, and contain contractile actomyosin, characteristics of mechanical function. Apical bulkheads persist at high pressure upon microinjection of fluid into the BC lumen, and laser ablation demonstrated that they are under tension. A mechanical model based on ablation results revealed that apical bulkheads double the pressure BC can hold. Apical bulkhead frequency anticorrelates with BC connectivity during mouse liver development, consistent with predicted changes in biliary pressure. Our findings demonstrate that apical bulkheads are load-bearing mechanical elements that could protect the BC network against elevated pressure.

## Teaser

Bebelman and Bovyn et al. demonstrate that apical bulkheads, connections between the apical membranes of opposing hepatocytes, are load-bearing mechanical elements that contribute to the ability of bile canaliculi to withstand elevated luminal pressure.

## Introduction

Hepatocytes are specialized polarized cells that form a network of bile canaliculi (BC) with their apical surfaces, enabling secretion and transport of bile (Treyer and Müsch, 2013). During hepatoblast-to-hepatocyte differentiation in the developing liver, adjacent hepatoblasts initially form small spherical lumina between their apical membranes. These apical lumina then expand anisotropically, generating narrow tubules that ultimately connect to complete the formation of the BC network (Belicova et al., 2021). Similar to lumen expansion in other epithelia, BC elongation is driven by secretion of ions (bile acids and other organic solutes) into the nascent lumen (Boyer, 2013; Bryant and Mostov, 2008; Dasgupta et al., 2018). This generates an osmotic pressure that drives passive influx of water into the lumen and creates a hydrostatic pressure that induces lumen expansion. To achieve anisotropic expansion of the apical lumen, hepatocytes need to balance the isotropic hydrostatic pressure in the lumen with anisotropic forces in the subapical actomyosin cortex (Li et al., 2016). We recently reported that BC elongation is enforced by apical bulkheads, F-actin–containing extensions of the apical membrane that traverse the canalicular lumen and connect juxtaposed hepatocytes (Belicova et al., 2021). The structure of apical bulkheads and their role in BC formation suggest that they are mechanical elements that generate anisotropy in the subapical actomyosin cortex, thereby driving BC elongation.

As the liver develops and nascent BC connect to the expanding BC network, the hydrostatic pressure resulting from the secretion of bile acids and other solutes into the luminal space induces a flow of bile toward the bile duct (Boyer, 2013; Sperber, 1959). However, even in the mature liver, the pressure inside the biliary tree fluctuates as a consequence of increased bile acid clearance from the blood by hepatocytes following feeding (Angelin et al., 1982; Weis and Barth, 1978). Furthermore, impaired bile flow as a result of bile duct obstruction by gallstones or cholestatic liver disease increases the pressure in the biliary tree. Interestingly, we recently observed an accumulation of apical bulkheads in response to increased luminal pressure, both in a mouse model of bile-duct ligation and in tissues of patients with cholestatic liver disease (unpublished data). These findings suggest that in addition to their role in development, apical bulkheads may also act as mechanical elements that counteract physiological and pathological elevations of bile pressure.

In this study, we experimentally tested the function of apical bulkheads as mechanical elements that oppose canalicular bile pressure. As a model system, we used in vitro–differentiated hepatocytes that form elongated BC (Belicova et al., 2021). In this culture system, the BC remain closed, preventing the drainage of bile, reflecting the elevated bile pressure that hepatocytes experience during development and in cholestatic liver.

## Results

### Apical bulkhead halves are sealed by tight junction loops and connected by adherens junctions

**Figure 1.**
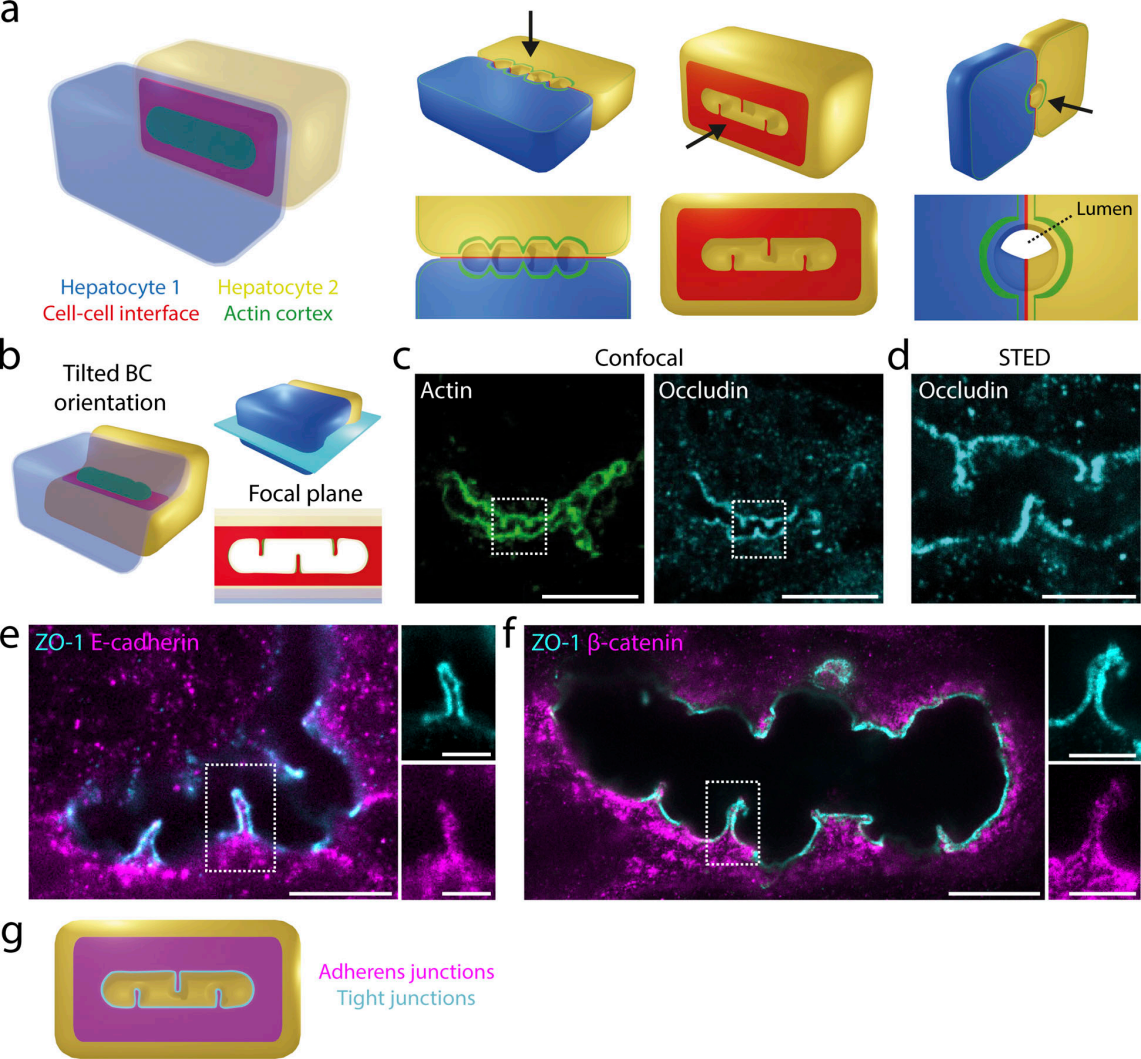
**Apical bulkhead halves are connected by adherens junctions and sealed by tight junction loops. (a)** Left: Schematic model of a BC formed by two adjacent hepatocytes (transparent blue and yellow) in the paradigmatic BC orientation. Right: Visualization of the BC with apical bulkheads from different angles (black arrows) after various cuts of the model (upper) with associated views (lower). **(b)** Imaging strategy used to visualize the intercellular junctions that connect apical bulkhead halves by STED microscopy. Left: Schematic model of a BC that is tilted 90° compared with the paradigmatic BC orientation. Upper right: The focal plane (light blue rectangle) used for STED microscopy. Lower right: The microscopy view at the cell–cell interface between apical bulkhead halves. **(c)** Confocal microscopy of a tilted BC between two hepatocytes in vitro, stained for F-actin with phalloidin–AlexaFluor-488 and the tight junction protein Occludin. Scale bar: 10 μm. **(d)** STED microscopy of Occludin corresponding to the region highlighted in Fig. 1 c. Scale bar: 2 μm. **(e and f)** STED microscopy of tilted BC between two hepatocytes in vitro stained for the tight junction protein ZO-1 and the adherens junction proteins E-cadherin (e) and β-catenin (f). Scale bars: 5 μm (overview) and 2 μm (zoom). **(g)** Schematic representation of the tight junctions (cyan) and adherens junctions (magenta) connecting apical bulkhead halves.

**Figure S1.**
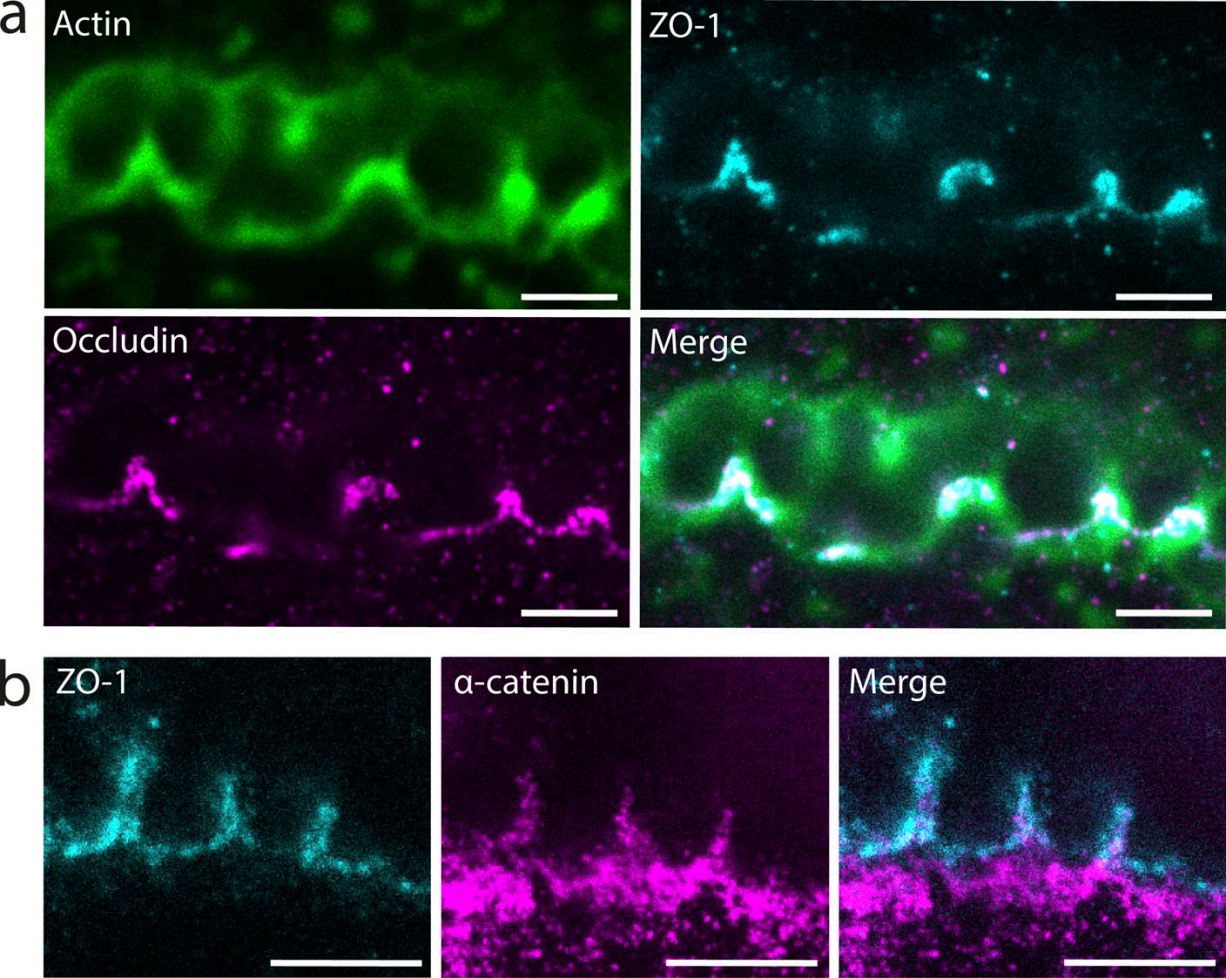
**Apical bulkhead halves are connected by adherens junctions and sealed by tight junction loops. (a)** Confocal and STED microscopy of a tilted BC between two hepatocytes in vitro stained for F-actin with phalloidin–AlexaFluor-488 (confocal) and the tight junction proteins ZO-1 (STED) and Occludin (STED). Scale bar: 2 μm. **(b)** STED microscopy of a tilted BC between two hepatocytes in vitro stained for ZO-1 and the adherens junction protein ⍺-catenin. Scale bar: 2 μm.

Apical bulkheads consist of two halves that protrude from the apical membranes of opposing hepatocytes into the canalicular lumen (Fig. 1 a; Belicova et al., 2021). Using immunofluorescence and electron microscopy analysis, we previously found that apical bulkhead halves are connected by unusual T-shaped intercellular junctions perpendicular to the junctions that are longitudinal along the BC (Belicova et al., 2021). To understand these structures in more detail, we visualized them using stimulated emission depletion (STED) super-resolution microscopy. In the paradigmatic BC orientation with the plane between the two opposing apical surfaces perpendicular to the adhesion substrate (Fig. 1 a), the intercellular junctions that connect the apical bulkhead halves are oriented along the optical axis of the microscope, limiting the maximum resolution that can be achieved. To circumvent this problem, we specifically imaged BC that were tilted close to 90° as compared with the paradigmatic BC orientation (Fig. 1 b). Such tilted BC orientation can frequently be observed in vitro and, depending on the plane of the section, in liver tissue (Belicova et al., 2021). We visualized cells stained for F-actin and the tight junction proteins Occludin and ZO-1 by confocal (Fig. 1 c) and STED super-resolution microscopy (Fig. 1 d and Fig. S1 a). We observed that the tight junctions form a narrow loop that lines the surface connecting two apical bulkhead halves and is continuous with the tight junction lining the BC (Fig. 1 d and Fig. S1 a). Furthermore, the adherens junction proteins E-cadherin and α- and β-catenin all localize to the cell–cell interface between apical bulkhead halves (Fig. 1, e and f; and Fig. S1 b). These results indicate that opposing apical bulkhead halves are sealed by tight junction loops and connected by adherens junctions (Fig. 1 g). The presence of adherens junctions suggests that apical bulkheads could be load-bearing structures that mechanically connect the apical membranes of opposing hepatocytes.

### Apical bulkheads contain a contractile actomyosin cytoskeleton

**Figure 2.**
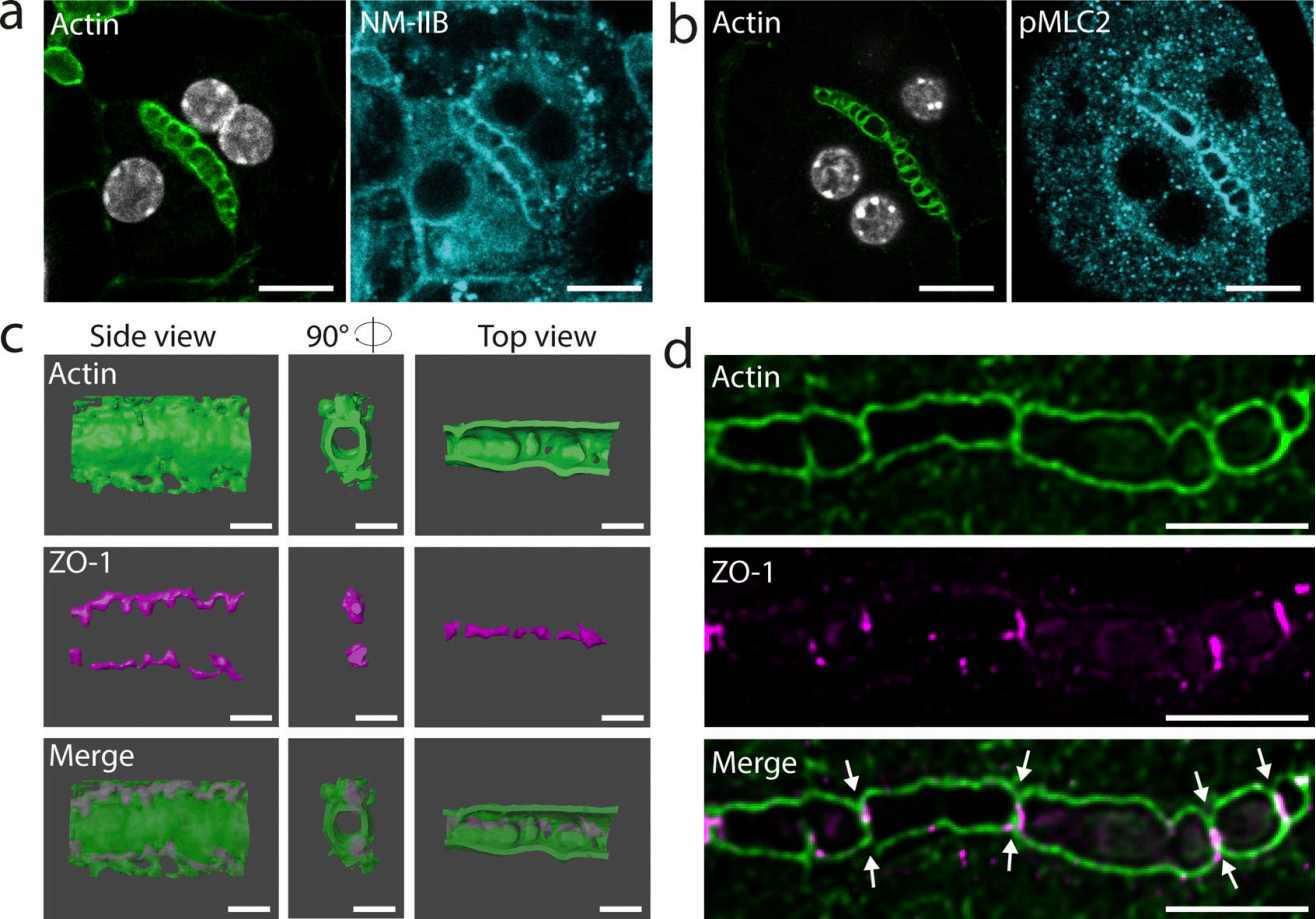
**Apical bulkheads contain a contractile actomyosin cytoskeleton. (a and b)** In vitro–differentiated hepatocytes stained for F-actin with phalloidin–AlexaFluor-488 and for non-muscle myosin IIb (a) or phosphomyosin light chain 2 (Ser19; b). Scale bars: 10 µm. **(c)** 3D reconstruction of the actin cortex (phalloidin, green) and tight junctions (ZO-1, magenta) in a BC segment that was imaged by 3D-STED microscopy (see also Video 1). Scale bar: 2 µm. **(d)** Single z-slice of a BC segment stained for F-actin with phalloidin–STAR635 and the tight junction marker ZO-1 stained by 3D-STED microscopy. White arrows indicate positions where the subapical actomyosin cortex evaginates into the BC, forming apical bulkhead halves that are sealed by tight junctions. Scale bar: 5 µm.

If apical bulkheads are mechanical elements, one can expect them to contain a contractile actomyosin cytoskeleton. To test this hypothesis, we first assessed the presence of molecular markers diagnostic of actomyosin contractility. Indeed, besides F-actin, we found that apical bulkheads contain non-muscle myosin 2B (Fig. 2 a). Furthermore, the bulkhead myosin is active, as indicated by the presence of phosphorylated myosin light chain (Fig. 2 b). Next, we aimed to understand whether the bulkhead actomyosin is part of the apical actomyosin cortex or forms a separate actomyosin structure. To this end, we investigated the structural organization of the actomyosin cytoskeleton in apical bulkheads using 3D-STED microscopy which enables imaging with isotropic resolution (Fig. 2 c and Video 1). We observed that the bulkhead actomyosin cytoskeleton is continuous with the rest of the apical actomyosin cortex (Fig. 2 d). These results suggest that apical bulkheads contain a contractile actomyosin cortex and support the hypothesis that apical bulkheads act as mechanical elements.

**Video 1.** **3D-STED microscopy of actin (phalloidin, green) and tight junctions (ZO-1, magenta) in a BC segment in hepatocytes in vitro (the same BC segment that is reconstructed in** Fig. 2 c**)**.

### Apical bulkhead structure persists at high luminal pressure

**Figure 3.**
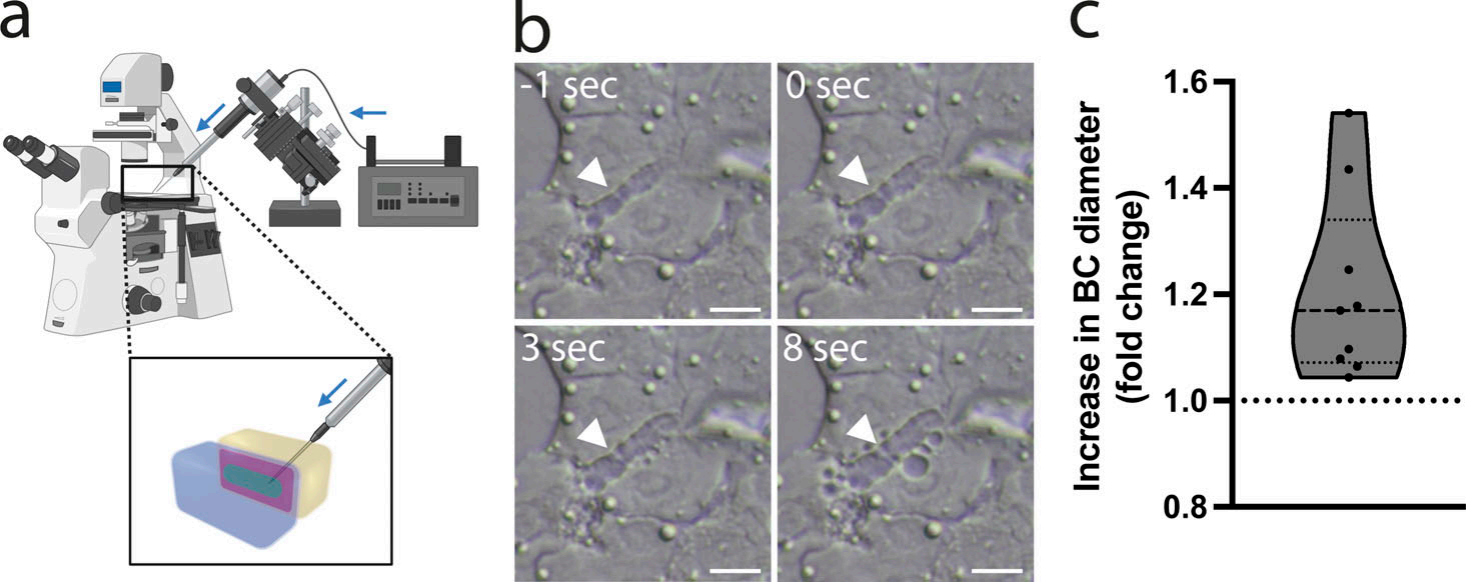
**Apical bulkhead structure persists at high luminal pressure. (a)** Schematic representation of the microinjection setup used to acutely raise intraluminal pressure in the BC. Blue arrows indicate direction of flow. Created with BioRender.com. **(b)** Still images from live-cell time-lapse brightfield microscopy before and during microinjection of fluid into a BC (see also Video 2). White arrowhead indicates the position of an apical bulkhead. Scale bar: 10 µm. **(c)** Quantification of the fold increase in BC diameter before microinjection of fluid into BC causes blebbing of the apical membrane into the cytoplasm (*n* = 9, obtained in three independent experiments).

The apical bulkheads in developing liver have been proposed to provide mechanical stability to the elongating BC lumen under inner pressure (Belicova et al., 2021). To facilitate BC elongation, apical bulkheads need to persist when luminal pressure rises and tension in the actomyosin cortex increases. To assess the effect of elevated pressure on apical bulkheads, we used a microinjection setup to inject fluid into the BC, thereby raising luminal pressure acutely (Fig. 3 a). We observed that microinjection initially caused dilation of the BC and ultimately resulted in blebbing of the apical membrane into the cytoplasm (Fig. 3 b and Video 2). Such inward blebs have previously been described in cholestatic BC and result from ruptures in the apical actomyosin cortex as a consequence of high luminal pressure (Gupta et al., 2017). To assess the stretching ability of the BC actomyosin cortex, we measured the diameter increase during microinjection and found a mean BC diameter increase of ∼20% by the time the first inward bleb occurs (Fig. 3 c). Importantly, increasing the pressure in the BC by microinjection never resulted in the loss of apical bulkheads, even at pressures at which inward blebbing occurs (Fig. 3 a). This reveals that apical bulkheads are more resistant to acute elevated pressure than the rest of the apical actomyosin cortex, which is consistent with their hypothesized mechanical role in cholestasis.

**Video 2.** **Time-lapse live-cell brightfield microscopy showing microinjection of fluid into a BC in vitro (video associated with** Fig. 3 b**).** Images acquired at 10 Hz, and the video plays at 1× normal speed.

### Tension over apical bulkheads supports BC shape

**Figure 4.**
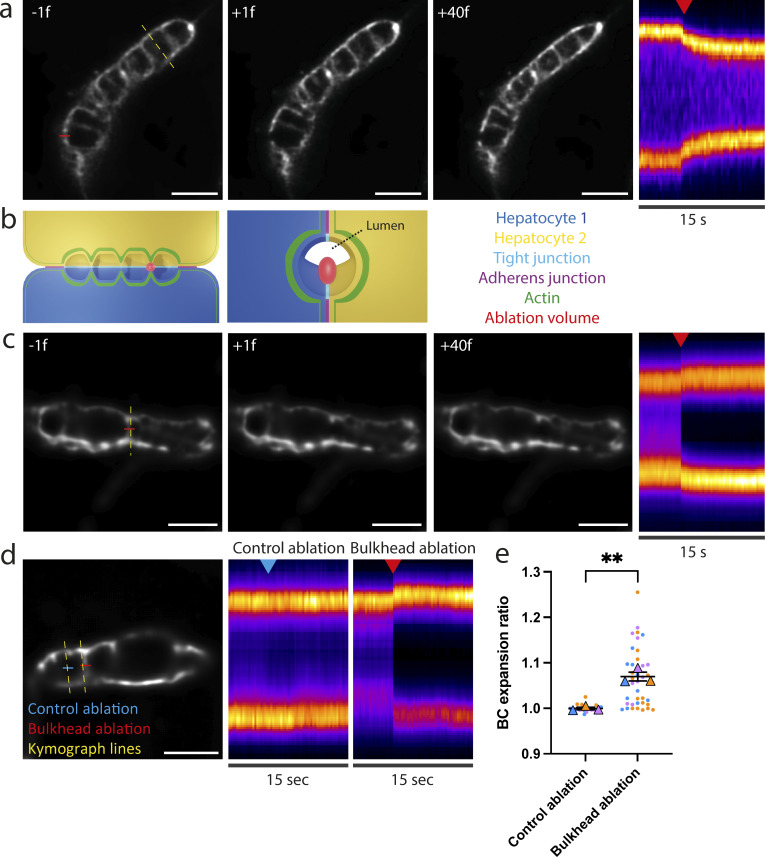
**Apical bulkheads are under tension. (a)** Left: Still images from a BC labeled with SiRactin the frame before (−1f), the frame directly after (+1f), and 40 frames after (+40f) ablation of the apical membrane (see also Video 3). Ablation line is indicated in red, kymograph line is indicated in yellow. Scale bar: 5 µm. Right: Kymograph revealing BC deflation following ablation of the apical membrane. Red arrowhead indicates moment of laser ablation. **(b)** Schematic representation of the laser ablation of individual apical bulkheads. Expected ablation volume in red. **(c)** Left: Still images from a BC labeled with SiRactin the frame before (−1f), the frame directly after (+1f), and 40 frames after (+40f) ablation of an individual apical bulkhead (see also Video 4). Ablation line is indicated in red, kymograph line is indicated in yellow. Scale bar: 5 µm. Right: Kymograph revealing an immediate local expansion of the BC following ablation of the apical bulkhead. Red arrowhead indicates moment of laser ablation. **(d)** Left: Still image from a BC labeled with SiRactin before control ablation next to an apical bulkhead and laser ablation of an apical bulkhead. Scale bar: 5 µm. Right: Kymographs revealing that only ablations of apical bulkheads, but not control ablations, causes an immediate local BC expansion. Red and blue arrowheads indicate the moment of laser ablation. **(e)** Quantification of local BC expansion upon laser ablation of apical bulkheads (*n* = 38) and control laser ablations (*n* = 21), obtained in three independent experiments. Data are color-coded by biological replicate, where dots represent individual ablations, triangles represent the mean of each biological replicate, and the horizontal bars represent the average of the means ± SEM. **, P < 0.01 using an unpaired Student’s two-tailed two-sample *t* test of the means of each biological replicate.

**Figure S2.**
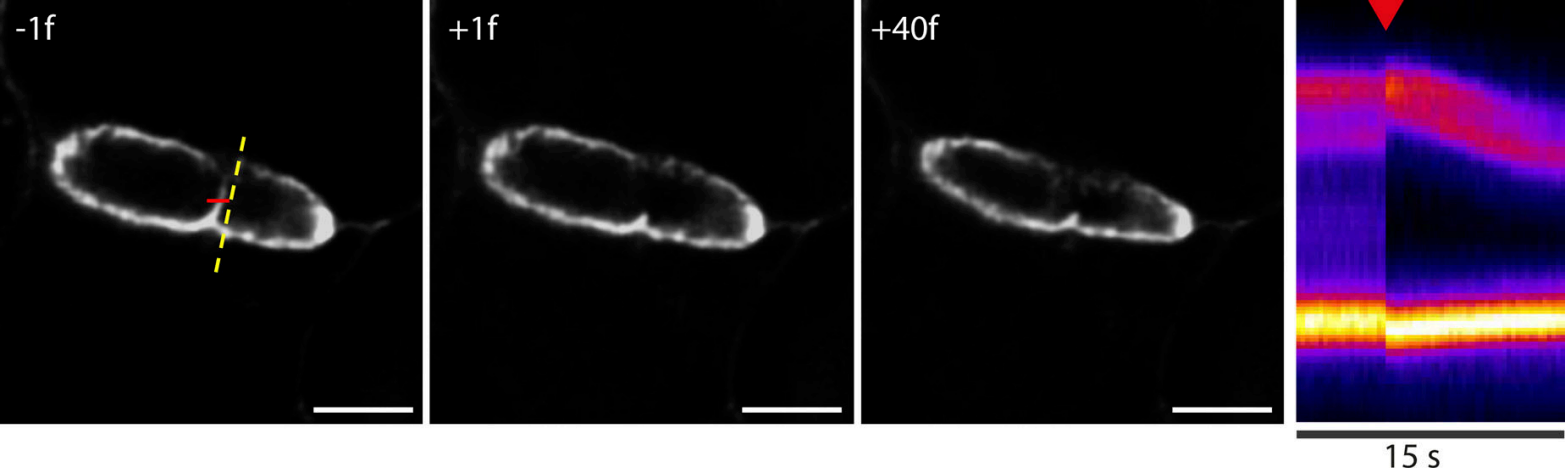
**Laser ablation of an apical bulkhead. **Left: Still images from a BC labeled with SiRactin the frame before (–1f), the frame directly after (+1f), and 40 frames after (+40f) ablation of an individual apical bulkhead (see also Video 5). Ablation line is indicated in red, kymograph line is indicated in yellow. Scale bar: 5 µm. Right: Kymograph revealing an immediate local expansion of the BC following ablation of the apical bulkhead and subsequent BC deflation. Red arrowhead indicates moment of laser ablation.

The presence of contractile actomyosin and adherens junctions in apical bulkheads suggests that they are mechanical elements that oppose bile pressure. To provide direct evidence for this hypothesis, we set out to measure the effect of laser ablation of apical bulkheads on BC shape. As a control, we first ablated the apical membrane away from bulkheads. Not surprisingly, puncturing the hepatocyte apical membrane results in deflation of the BC, demonstrating that the pressure inside the BC is higher than in the cytoplasm (Fig. 4 a and Video 3). We then proceeded to ablate individual apical bulkheads while taking care to limit damage to the rest of the apical membrane (Fig. 4 b). To achieve this, we used an 800-nm fs pulsed ablation laser that enables ablations with a high axial resolution due to nonlinear absorption. Furthermore, we reduced damage to the base of the bulkhead by placing the ablation focal plane at the ridge of the bulkhead that is farthest inside the BC (Fig. 4 b). This strategy enabled us to ablate apical bulkheads without noticeable BC deflation in ∼42% of ablations. Following ablation, we observed immediate expansion of the BC surrounding the ablated apical bulkhead (within one frame of 250 ms), suggesting that it was under tension (Fig. 4 c and Video 4). This immediate local expansion could also be observed for ablation attempts that ultimately resulted in BC deflation (Fig. S2 and Video 5). To show that this immediate BC expansion is caused by the ablation of apical bulkheads, and not due to shock waves or other direct effects of the laser pulse, we performed control ablations in the BC lumen away from bulkheads. Although control ablations did not affect BC diameter, ablation of apical bulkheads caused an immediate local BC expansion in ∼57% of ablation attempts (Fig. 4, d and e). We suspected that the lack of immediate BC expansion in the remaining ablation attempts could result from insufficient axial precision in the positioning of the ablation volume, causing incomplete bulkhead ablation or fast BC deflation. Indeed, in some cases, a second ablation attempt sufficed to induce local BC expansion after a previous unsuccessful ablation. In conclusion, ablation of apical bulkheads frequently results in immediate local BC expansion, demonstrating that bulkheads are under tension and act as mechanical elements that hold together the apical membranes of juxtaposed hepatocytes and oppose bile pressure.

**Video 3.** **Time-lapse live-cell imaging showing laser ablation of the BC apical surface of SiRactin labeled hepatocytes in vitro, followed by BC deflation (video associated with** Fig. 4 a**).** Images acquired at 4 Hz, and the video plays at 5× normal speed. Scale bar: 5 µm.

**Video 4.** **Time-lapse live-cell imaging showing laser ablation of an apical bulkhead of SiRactin-labeled hepatocytes in vitro, followed by immediate local expansion of the BC (video associated with** Fig. 4 c**)**. Images acquired at 4 Hz, and the video plays at 5× normal speed. Scale bar: 5 µm.

**Video 5:** **Time-lapse live-cell imaging showing laser ablation of an apical bulkhead of SiRactin-labeled hepatocytes in vitro, followed by immediate local expansion of the BC and subsequent BC deflation (video associated with** Fig. S2**).** Images acquired at 4 Hz, and the video plays at 5× normal speed. Scale bar: 5 µm.

### Apical bulkheads increase the ability of the BC to hold pressure

**Figure 5.**
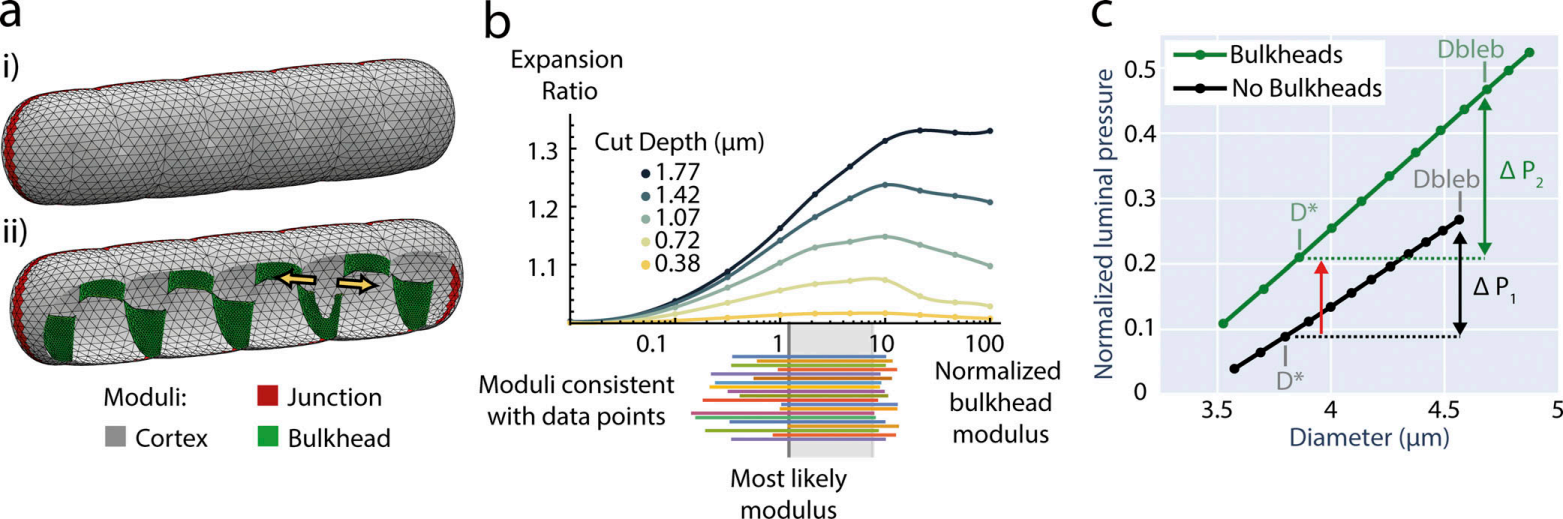
**Apical bulkheads increase the ability of BC to hold pressure. (a)** Mechanical model of a BC. Colored regions have different elastic moduli. **(i)** Outside view. **(ii)** Inside view with one bulkhead ablated, as indicated by the yellow arrowheads. **(b)** Simulated expansion ratios as a function of apical bulkhead modulus and ablation depth (curves inside axes). For each experimentally measured expansion ratio, minimum and maximum ablation depths yield maximum and minimum inferences on apical bulkhead modulus (below axes). There is a range of moduli consistent with all data (gray region below axis). In this range, the lowest modulus is the most likely (below axis, dark gray line). Bulkhead modulus (x-axis) is normalized to the modulus of the cortex. **(c)** The pressure at which BC with apical bulkheads have circular cross section (D*) is about double the pressure of equivalent BC with no bulkheads (red arrow). The increase in pressure before the critical BC diameter Dbleb reached is about 35% greater in BC with apical bulkheads (ΔP_2_) than without (ΔP_1_).

**Figure S3.**
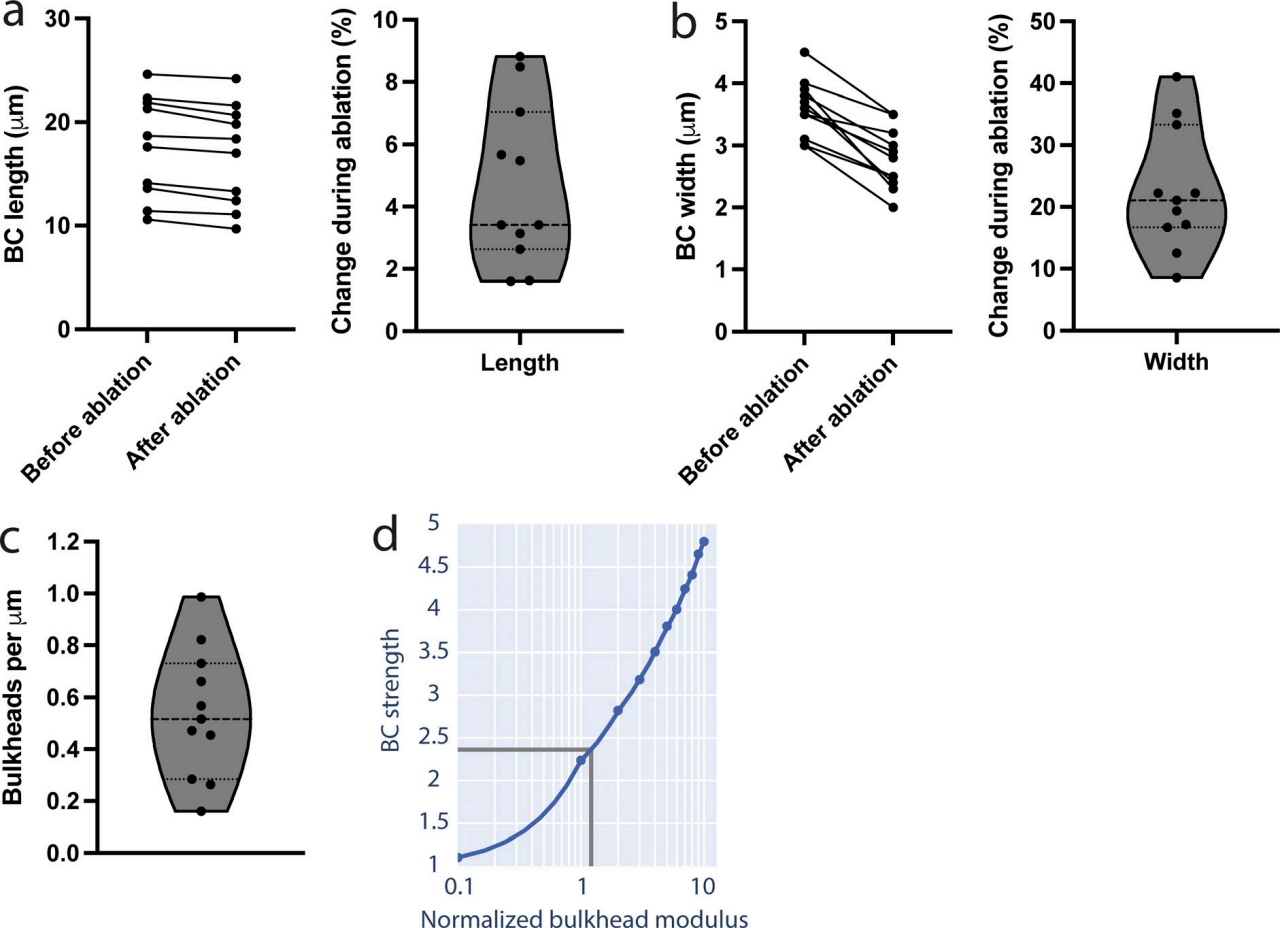
**Measurements for BC shape before and after deflation that were used to parameterize the mechanical BC model. (a–c)** (a) Length, (b) width, and (c) the number of bulkheads measured. **(d)** Increase in pressure held by BC before exceeding circular cross-section (BC strength, y-axis) with bulkheads as a function of the bulkhead modulus (x-axis, normalized to the modulus of the cortex).

The laser ablation experiments show that apical bulkheads are mechanical elements, suggesting that they contribute to the ability of the BC to hold pressure. It is currently not possible to measure pressure inside the thin BC lumen directly. However, the quantitative measurements of the laser ablation experiments raise the possibility that the contribution of apical bulkheads to the ability to hold pressure can be inferred through a mechanical model. Therefore, we developed such a model matching the 3D shape of BC and parameterized it using the experimentally measured BC deflation and bulkhead ablation data of Fig. 4. To assess the extent to which apical bulkheads contribute to the ability of the BC to hold pressure, we compared BC models with and without bulkheads. The elastic response of the cortex dominates at the timescale of the laser ablation experiments (sub-second), given that the turnover of actin (∼30 s) and adherens junctions (minutes) is much slower (de Beco et al., 2009; Salbreux et al., 2012). Therefore, we modeled the BC structure as an elastic surface. An elastic surface has a preferred shape, and when stretched, it exerts a force to return to that shape. The magnitude of this force is proportional to the elastic modulus, which is a measure of the ability of a material to resist a deforming force. The equations we used to describe the mechanics of the elastic surface are detailed in Appendix Section 1. We solved these equations using the programming language Surface Evolver (Brakke, 1992), where we created a 3D model of the BC with apical bulkheads (Fig. 5 a, i and ii). The 3D BC model was defined by parameters of shape (length, width, etc.) and mechanics (elastic moduli and luminal pressure). To reduce the complexity of the model, apical bulkheads were modeled as single structures, which encompass both bulkhead halves connected by tight and adherens junctions. The shape parameters were set to match measurements of BC before and after laser ablation–induced BC deflation to get the expanded and elastically preferred BC shapes, respectively (Fig. 4 a and Fig. S3, a–c; Appendix Section 2).

We modeled the mechanics of the BC by imposing different elastic moduli for (1) the surrounding cortex, (2) junctions, and (3) bulkheads (Fig. 5 a, i and ii). The junction modulus and bulkheads modulus were normalized to the surrounding cortex modulus. The junction modulus was determined by matching shapes before and after deflation (Fig. S3, a–c; Appendix Section 2). To estimate the bulkheads modulus, we simulated laser ablations and compared the results with the experimental data (Fig. 5 a, ii; Appendix Section 3). Since the precise amount of bulkhead removed in each experimental ablation is unknown, we simulated a range of cut depths (Fig. 5 b). Obviously, the expansion after ablation is determined by both the bulkhead modulus and the depth of the cut. Therefore, one might think that the bulkhead modulus cannot be determined from the measured expansion ratios, given the variability of cut depth in each ablation. However, we found that for each cut depth, there is a maximum expansion ratio with respect to the elastic modulus of the apical bulkheads (see Appendix Section 3B for elaboration). This implies that there is only a limited range of bulkhead elastic moduli, between 1.2- and 10-fold the cortex elastic modulus, that is compatible with the experimental data (Fig. 5 b). From this range, the lowest value of 1.2 is the most likely (see Appendix Section 3D). This is consistent with our observation that the actomyosin cortex in the apical bulkheads is continuous with and similar to the rest of the apical cortex (Fig. 2 d).

With this parameterized model, we investigated the contribution of the apical bulkheads to the mechanical structure of the BC by comparing simulations of BC with and without bulkheads. In experiments, we observed that under elevated luminal pressure, BC remain roughly circular in cross-section, i.e., do not excessively bulge out into the cytoplasm (Fig. 2 c). In our model, BC with bulkheads can accommodate twice the pressure of BC without bulkheads without exceeding circular cross section (denoted by the red arrow in Fig. 5 c). In the following, we term the pressure BC can hold before exceeding circular cross-section as “strength” and conclude that BC with bulkheads are twice as strong. We explored the sensitivity of our inference about BC strength by examining it over a range of bulkhead moduli (Fig. S3 d). We find that BC strength is qualitatively similar when apical bulkheads have elastic moduli in the range from about 0.5 to 2 times the inferred value of 1.2. Inspired by our microinjection experiments, we also determined the additional pressure that BC can bear before expanding beyond the limit at which blebbing occurs (∼20% increase in diameter, Fig. 3 c). BC with bulkheads can bear ∼35% more pressure increase than BC without bulkheads before this limit (Fig. 5 c). This result suggests that apical bulkheads also contribute to avoiding ruptures in the cortex caused by acute elevations of pressure.

### Apical bulkhead frequency anticorrelates with BC connectivity in the developing liver

**Figure 6.**
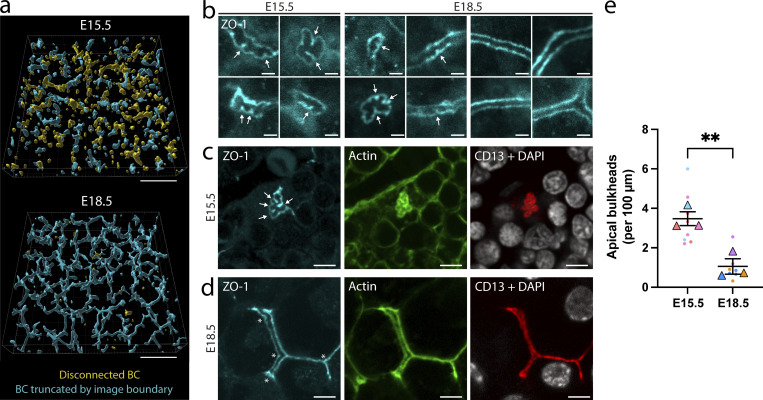
**Apical bulkhead frequency anticorrelates with BC connectivity. (a)** Reconstruction of the BC network in embryonic liver tissue at E15.5 (upper) and E18.5 (lower) based on CD13 staining. Individual BC fragments are classified based on whether they are disconnected from the rest of the network (yellow) or truncated by the boundaries of the imaging volume (blue). Scale bar: 50 µm. **(b)** Examples of BC stained for the tight junction protein ZO-1 (cyan) containing apical bulkheads at E15.5 (left four panels) and E18.5 (middle four panels), as well as the typical BC morphology without apical bulkheads at E18.5 (right four panels). White arrows indicate apical bulkheads. Scale bar: 2 µm. **(c)** Example of a disconnected BC from E15.5 with apical bulkheads (white arrows). Liver tissue was stained for ZO-1 (cyan), actin (green), the apical marker CD13 (red), and DAPI (white). Scale bar: 5 µm. **(d)** Example of a BC from E18.5 without apical bulkheads. Liver tissue was stained for ZO-1 (cyan), actin (green), the apical marker CD13 (red), and DAPI (white). Asterisks indicate branching points in the BC network. Scale bar: 5 µm. **(e)** Quantification of the number of apical bulkheads in embryonic liver tissue at E15.5 and E18.5, normalized to BC length. Data is color-coded by biological replicate, dots represent the normalized apical bulkhead frequency in a single imaging volume, triangles represent the mean of each biological replicate imaged at 2–3 locations per liver, and the horizontal bars represent the average of the means ± SEM. **, P < 0.01 using an unpaired Student’s two-tailed two-sample *t* test of the means of each biological replicate.

The ability of apical bulkheads to increase BC strength suggests that they are especially needed when the pressure inside the canalicular lumen is high. To test this hypothesis, we compared the number of apical bulkheads at early and late stages of BC network development in mice. At E15.5 (15.5 d after conception), nascent BC have formed, but the majority is not yet connected to bile ducts (Fig. 6 a). This prevents bile from draining away and results in elevated bile pressure, which in turn drives lumen expansion (Boyer, 2013; Dasgupta et al., 2018). At E18.5, the BC network is almost fully connected (Fig. 6 a) and the bile can drain via the bile ducts into the intestines (Tanimizu et al., 2016), causing a drop in canalicular pressure. Therefore, we would predict that at E15.5, the frequency of apical bulkheads is higher than at E18.5. To visualize apical bulkheads, we performed deep tissue imaging on liver sections stained for ZO-1, actin, and the apical marker CD13. Apical bulkheads were identified based on the presence of sharp protrusions of the tight junction into the BC lumen, as in Fig. 1, c–f (Fig. 6 b; see Materials and methods for details). At E15.5, we could observe many apical bulkheads in nascent BC that were not yet connected to the network (Fig. 6, b and c). At E18.5, we could occasionally find apical bulkheads (Fig. 6 b), but the majority of BC had straight and parallel tight junctions that did not have protrusions into the BC lumen (Fig. 6, b and d). We quantified the number of apical bulkheads per unit of BC length (see Materials and methods) and found that, consistent with our hypothesis, they were more frequent at E15.5 compared with E18.5 (Fig. 6 e). Moreover, 44% of the apical bulkheads observed at E18.5 were located in disconnected BC, even though these only account for a small fraction of the total BC network (yellow lumina in Fig. 6 a, lower panel). These results show that BC connectivity and apical bulkhead frequency are anticorrelated and suggest that apical bulkheads protect nascent BC from high bile pressure before they connect to the BC network and bile pressure drops.

## Discussion

In this study, we have experimentally tested the hypothesis that hepatocyte apical bulkheads are mechanical elements that oppose canalicular bile pressure. Morphological characterization by super-resolution STED microscopy revealed that apical bulkheads contain molecular components (contractile actomyosin cortex and adherens junctions) that enable them to bear a mechanical load. Using a laser ablation approach, we directly demonstrated that hepatocyte apical bulkheads are under tension and mechanically connect the apical membranes of opposing hepatocytes. We generated a parametrized mechanical BC model and determined that apical bulkheads double the pressure that BC can hold. Finally, we found a negative correlation between apical bulkhead frequency and BC connectivity during liver development, suggesting that apical bulkheads contribute to the protection of nascent BC against high canalicular pressure.

How apical bulkheads are formed is an outstanding question. The tight junction loops that we observed by STED microscopy, as well as the organization of actomyosin cortex and the presence of adherens junctions, suggest that apical bulkheads are formed by folding the intercellular junction line into the apical lumen. Folds in tight junctions have previously been demonstrated in columnar epithelia (Fanning et al., 2002; Shen et al., 2006). However, in the case of apical bulkheads, the tight junctions are bent in such a way that the apical surface folds inside the BC. F-actin is enriched along the tight junction in comparison with the rest of the apical cortex (Belicova et al., 2021; Porat-Shliom et al., 2016). It is possible that such inhomogeneity in actomyosin meshwork, in combination with increased actomyosin contractility in response to pressure (Gupta et al., 2020; Sethi et al., 2017), generates a disbalance in tension, resulting in folds of the apical surface centered around the tight junctions. In this case, the height of the apical bulkheads would depend on the luminal pressure. This interpretation would explain why the frequency of apical bulkheads is higher at early stages of BC network development when nascent BC are not yet connected to bile ducts.

Apical bulkheads can also be observed in adult liver (Belicova et al., 2021), albeit at a lower frequency than during early BC network development, suggesting that they have a physiological role in tissue homeostasis. Given our results, one attractive possibility is that they protect the BC network against fluctuations in biliary pressure, e.g., induced by food intake. Direct measurement of the pressure inside BC is currently not possible. However, measurements of bile acid clearance from portal venous blood by hepatocytes after feeding suggest a maximal increase in canalicular pressure up to threefold (Angelin et al., 1982; Voronova et al., 2020). In cholestatic liver diseases, and in particular upon complete bile duct obstruction, pressure in the canaliculi increases (Gupta et al., 2017). To maintain BC network integrity, hepatocytes respond by decreasing bile acid synthesis and strengthening the subapical actomyosin cortex (Meyer et al., 2020; Song et al., 1996; Zollner et al., 2006). Interestingly, apical bulkheads accumulate under cholestatic conditions, i.e., in hepatocytes in vitro (Belicova et al., 2021), upon bile duct ligation in mice, and in livers of patients with cholestatic liver disease (unpublished data), suggesting that apical bulkheads may act as a mechanical response to elevated bile pressure. Based on a pressure gradient of one order of magnitude between BC and bile duct in physiology (Meyer et al., 2017) and a 12.5-fold increase in bile duct pressure following bile duct ligation (Wiener et al., 2000), one can expect that complete obstruction of bile flow increases canalicular pressure by less than one order of magnitude. As such, a twofold increase in BC strength mediated by apical bulkheads is likely to contribute significantly to the protection of the BC network against physiological and pathological elevations of luminal pressure.

## Materials and methods

### Hepatoblast isolation and in vitro differentiation toward hepatocytes

Hepatoblast isolation from embryonic livers (E13.5) of C57BL/6JOlaHsd mice and subsequent in vitro differentiation toward hepatocytes was performed as described in Belicova et al. (2021). In brief, embryonic livers were fragmented in Liver Perfusion Medium (cat. no. 17701–038; Thermo Fisher Scientific), followed by digestion in Liver Digest Medium (cat. no. 17703–034; Thermo Fisher Scientific) supplemented with 10 µg/ml DNase I (cat. no. DN25; Sigma-Aldrich,). Erythrocytes were lysed in red blood cell lysis buffer: 155 mM NH_4_Cl, 10 mM KHCO_3_, and 0.1 mM EDTA, pH 7.4. After blocking of the digested cells with anti-mouse CD16/CD32 blocking antibody (cat. no. 553142; Rat, BD Biosciences), Dlk1+ hepatoblasts were labeled with anti-Dlk mAb-FITC (cat. no. D187-4; Rat, MBL) and anti-FITC MicroBeads (cat. no. 130–048-701; Miltenyi Biotec). Labeled Dlk + hepatoblasts were isolated using a magnetic column (cat. no. 130–024-201; Miltenyi Biotec) according to the manufacturer’s protocol. Isolated hepatoblasts were seeded on Matrigel-coated CellView slides (cat. no. 543979; Greiner) at a density of 13,000 cells/well for microscopy and laser ablation or on 11-mm round glass coverslips in a 24-well plate at a density of 65,000 cells/well for microinjection in expansion media: DMEM/F-12, GlutaMAX supplement (cat. no. 31331028; Thermo Fisher Scientific), 10% FBS, 1 × insulin-transferrin-selenium-ethanolamine (cat. no. 51500–056; Gibco), 0.1 µM dexamethasone (cat. no. D1756-25 MG; Sigma-Aldrich), 10 mM nicotinamide (cat. no. N0636-100 G; Sigma-Aldrich), 10 ng/ml human hepatocyte growth factor (in-house production), and 10 ng/ml mouse epidermal growth factor (in-house production). After 24 h, the cells were overlaid with differentiation media: MCDB131 (cat. no. 10372019; Thermo Fisher Scientific), 2 mM L-glutamine (cat. no. M11-004; Thermo Fisher Scientific) 0.25 × insulin-transferrin-selenium-ethanolamine, and 0.1 µM dexamethasone, containing Matrigel (cat. no. 356231; Corning) to a final concentration of 5% vol/vol. Cells were cultured for 5 d in a humidified incubator with 5% CO_2_ at 37°C.

### Immunofluorescence staining

5 d after plating, hepatocytes were fixed with 3% PFA in PBS for 20 min at RT and washed with PBS before permeabilization and blocked using blocking buffer (2% BSA and 0.05% saponin in PBS) for 1 h at RT. Cells were incubated overnight at 4°C with primary antibodies diluted in a blocking buffer. Primary antibodies used were ZO-1 (cat. no. 40-2200; Rabbit; Invitrogen), ZO-1 (cat. no. 339100; Mouse; Invitrogen), E-cadherin (cat. no. 610181; Mouse; BD Biosciences), Occludin (cat. no. 71-1500; Rabbit; Thermo Fisher Scientific), β-catenin (cat. no. 610153; Mouse; BD Biosciences), ⍺-catenin (cat. no. ab51032; Rabbit; Abcam), nonmuscle myosin IIb (cat. no. PRB-445P; Rabbit; Covance), pMLC2-ser19 (cat. no. 3671; Rabbit; Cell Signaling). Phalloidin-488 (cat. no. A12379; Thermo Fisher Scientific), Phalloidin-STAR-635 (cat. no. ST635-0100; Abberior), and DAPI were included in the primary antibody solutions. After washing with PBS, cells were incubated with secondary antibodies diluted in a blocking buffer for 2 h at RT. Secondary antibodies used for STED microscopy were Goat Anti-Rabbit-StarRed (Abberior, cat. no. STRED-1002) and Goat Anti-Mouse-AlexaFluor-594 (cat. no. A11032; Thermo Fisher Scientific). Secondary antibody used for confocal microscopy was Goat Anti-Rabbit-AlexaFluor-647 (cat. no. A21244; Thermo Fisher Scientific).

### Confocal and STED microscopy of hepatocytes in vitro

Confocal microscopy was performed using an LSM700 inverted laser scanning confocal microscope (Zeiss) equipped with a 40×x/1.2 C-Apochromat, water, DIC objective (Zeiss), and Zen imaging software (Zeiss). For 2D- and 3D-STED, cells were imaged using an Abberior 3D-2 Color-STED system (Abberior Instruments) with a 100× /1.35 NA silicon objective (Olympus). AlexaFluor-594 and Abberior-SR/635 were imaged with a pulsed laser at 560 and 640 nm, respectively, in combination with a 775 nm, 40 MHz pulsed depletion laser (Katana HP, 3W, 1 ns pulse duration, NKT Photonics). Imspector imaging software (Abberior Instruments) was used for STED microscopy.

STED images in Fig. 1 and Fig. S1 represent a single z-slice or an average of five z-slices within a z-range of 250 nm. 3D-STED images (Fig. 2 d and Video 1) were deconvolved with Huygens Software (Scientific Volume Imaging) using the following parameters: background: automatic estimation, estimate mode: lowest, area radius: 0.7, deconvolution algorithm: Classic maximum likelihood estimation, maximum iteration: 80, signal-to-noise ratio: 25, quality threshold: 0.05, iteration mode: optimized, brick layout: auto. Microscopy images were analyzed using Fiji (Schindelin et al., 2012). ClearVolume (Royer et al., 2015) was used for visualization of the 3D-STED microscopy in Video 1. For the 3D reconstruction of the BC in Fig. 2 d, we used the free software platform MotionTracking (http://motiontracking.mpi-cbg.de) to generate 3D triangle meshes for ZO-1 and actin. The meshes were created through a combination of intensity segmentation, edge filtering steps, and size filtering. Blender (http://www.blender.org) was used for visualization of the 3D triangle meshes.

### Quantification of apical bulkheads in embryonic liver tissue

Embryonic livers of C57BL/6JOlaHsd mice were immersion fixed using 4% PFA and 0.1% Tween-20 in PBS for 2 d at 4°C, and stored in PBS until processing. The livers were embedded in 4% agarose in PBS and cut into 100-µm-thick sections (for quantification of apical bulkheads) or 50-µm-thick sections (for high-resolution staining, Fig. 6, c and d) on a vibratome (Leica VT1200S). Tissue sections were permeabilized with 0.5% Triton X-100 in PBS for 1 h at RT. Primary antibodies against CD13 (cat. no. NB100-64843; Rat; Novus) and ZO-1 (cat. no. 40-2200; Rabbit; Invitrogen) were diluted in Tx buffer (0.2% gelatin, 300 mM NaCl, and 0.3% Triton X-100 in PBS) and incubated for 2 d at RT. After washing five times for 15 min with 0.3% Triton X-100 in PBS, the sections were incubated with secondary antibodies goat anti-rat-AlexaFluor-647 (cat. no. A21247; Thermo Fisher Scientific) and goat anti-rabbit-AlexaFluor-647 (cat. no. A11036; Thermo Fisher Scientific), DAPI and phalloidin–AlexaFluor-488 (cat. no. A12379; Thermo Fisher Scientific) for another 2 d. After washing five times for 15 min with 0.3% Triton X-100 in PBS and three times for 1 min with PBS. For the 100-µm-thick sections, we used SeeDB optical clearing (adapted from Ke et al., 2013). The optical clearing started by incubating the slices in 25% fructose for 4 h, continued in 50% fructose for 4 h, 75% fructose overnight, 100% fructose (100% wt/vol fructose, 0.5% 1-thioglycerol, and 0.1 M phosphate buffer, pH 7.5) for 6 h, and finally overnight in SeeDB solution (80.2% wt/wt fructose, 0.5% 1-thioglycerol, and 0.1 M phosphate buffer). 100-µm-thick sections were mounted in SeeDB solution and 50-µm-thick sections were mounted in Mowiol. 100-µm-thick sections were imaged using an LSM780 inverted laser scanning confocal microscope (Zeiss) equipped with a 63×/1.3 LCI Plan-Neofluar, W/Glyc, DIC objective (Zeiss), and Zen imaging software (Zeiss). 50-µm-thick sections were imaged using an inverted spinning-disc microscope (Andor) with a Yokogawa CSU-W1 scan head and iXon Ultra 888 Monochrome EMCCD camera (Andor), equipped with a 100×/1.35 UPLSAPO silicone objective (Olympus) using Andor iQ imaging software. The BC network was visualized using Imaris image analysis software (Oxford Instruments)–based apical membrane staining (CD13) that was segmented using a smoothing of 0.6 µm. BC were classified based on whether they contact the edge of the imaging volume.

Apical bulkheads were manually counted in BC for which two opposed tight junctions (ZO-1) were visible on a single confocal microscopy optical slice and are oriented parallel to the imaging plane. Apical bulkheads were identified based on protrusions of tight junction into the BC lumen. To distinguish apical bulkheads from small undulations in the tight junction, only protrusions for which the depth was more than the full width at half maximum were considered apical bulkheads. Branching points in the canalicular network were excluded from the analysis. The number of apical bulkheads was normalized to the total length of BC satisfying the criteria for bulkhead identification. Statistical analysis was performed using an unpaired Student’s two-tailed two-sample *t* test of the means of each biological replicate using GraphPad Prism version 9.0 (GraphPad software). Data distribution was assumed to be normal, but this was not formally tested.

### Microinjection

In vitro differentiated hepatocytes (3–4 d after plating) on glass coverslips were microinjected with 5 mM Tris, 0.1 mM EDTA, pH 7.6, at RT. Needles for microinjection were made from glass capillaries (Hervard Apparatus) using a needle puller (Sutter instrument; settings: Heat: 700, Pull: 150, Velocity: 100, Time: 150, resulting in a needle opening of around 600 nm). The microinjection setup consisted of a TransferMan 4r manipulator (Eppendorf) and a FemtoJet pump (Eppendorf) generating a continuous flow with a backpressure between 40 and 80 hPa. Microinjections were performed on an Axiovert 200 M microscope (Zeiss) equipped with an LD Achroplan 40×/0.6 Korr air objective (Zeiss) and imaged at 10 Hz using an a2A2590-60ucPRO camera (Basler) and Pylon Viewer imaging software.

### Laser ablation

In vitro differentiated hepatocytes (5 d after plating) in CellView slides were preincubated for 1 h at 37°C with 1 µM SiRactin (cat. no. SC001; Spirochrome) in combination with 10 µM Verapimil. Laser ablation experiments were performed using an inverted laser scanning confocal LSM 780 NLO microscope (Zeiss) equipped with a 40×/1.2 C-Apochromat, Water, DIC objective (Zeiss), and Zen imaging software (Zeiss). During imaging, cells were kept in a humidified incubation chamber at 37°C and 5% CO_2_. SiRactin signal was imaged using a HeNe 633 nm laser. Hepatocytes were imaged at 4 Hz using a 52 nm pixel size (field of view 512 × 512 pixels). Laser ablations were performed using a Chameleon Vision II Ti:Sapphire 800 nm fs pulsed laser (Coherent) at the lowest intensity (50% transmission) that gave reliable ablation, as determined by ablation of the BC apical surface and observing BC deflation. Images were denoised using DivNoising (Prakash et al., 2021). For this, a noise model was generated by bootstrapping with Noise2Void (Krull et al., 2019) as described in Prakash et al. (2021). Further image analysis was performed using FIJI.

### Quantification of BC shape parameters before and after laser ablation of the apical surface

Shapes of BC were quantified using z-stacks of BC before and after ablation of the apical membrane and cortex. BC length was measured using hand-drawn lines along the centerline of the BC. The z-slice where the BC appeared longest was used. The mean length of five drawn lines was taken. Width was measured at five locations about equally spaced along the length of the BC pre-ablation. At each location, we selected the z-slice where the BC was widest. The width after ablation was measured at the same five locations. Means of these five locations were taken. Apical bulkheads were counted by examining the BC both in z-slices and x-y projection (constructed by the Volume Viewer plugin in FIJI). Apical bulkheads were counted when they were identifiable in both views and projected more than one quarter of the way into the BC.

### Quantification of expansion ratios following laser ablation of apical bulkheads

Expansion ratios were determined by drawing a line adjacent to the bulkhead before ablation and measuring the diameter of the BC based on the intensity profile of that line in the frame before ablation and after ablation. To prevent effects of fluorophore bleaching on apparent BC width, the lines were placed so that the peak intensities after ablation were at least 90% of the peak intensities before ablation. Control ablations were performed in BC in places where there were no apical bulkheads. Statistical analysis of control and apical bulkhead ablations was performed using an unpaired Student’s two-tailed two-sample *t* test of the means of each biological replicate using GraphPad Prism version 9.0 (GraphPad software). Data distribution was assumed to be normal but this was not formally tested.

### Mechanical BC model

A detailed description of the mechanical BC model can be found in Appendix. Code for generating and analyzing the model can be found at http://github.com/mbovyn/bile-canaliculus-elastic.

### Online supplemental material

Video 1 shows 3D-STED microscopy of actin (phalloidin, green) and tight junctions (ZO-1, magenta) in a BC segment in hepatocytes in vitro (the same BC segment that is reconstructed in Fig. 2 c). Video 2 shows time-lapse live-cell brightfield microscopy showing microinjection of fluid into a BC in vitro (video associated with Fig. 3 b). Video 3 shows time-lapse live-cell imaging showing laser ablation of the BC apical surface of SiRactin-labeled hepatocytes in vitro, followed by BC deflation (video associated with Fig. 4 a). Video 4 shows the time-lapse live-cell imaging showing laser ablation of an apical bulkhead of SiRactin-labeled hepatocytes in vitro, followed by immediate local expansion of the BC (video associated with Fig. 4 c). Video 5 shows the time-lapse live-cell imaging showing laser ablation of an apical bulkhead of SiRactin labeled hepatocytes in vitro, followed by immediate local expansion of the BC and subsequent BC deflation (video associated with Fig. S2). Appendix is a detailed description of the mechanical BC model.

## Supplementary Material

Appendix is a detailed description of the mechanical BC model.
