# Supplementary material for: Hepatocyte apical bulkheads provide a mechanical means to oppose bile pressure
Source: J Cell Biol. 2023 Jan 30;222(4):e202208002. doi: 10.1083/jcb.202208002 (PMC9930133; doi:10.1083/jcb.202208002)
Supplement: Appendix — is a detailed description of the mechanical BC model. [file JCB_202208002_TextS1.pdf]

Appendix for
Hepatocyte apical bulkheads provide a mechanical means to oppose bile pressure

I. ELASTIC MODEL OF BILE CANALICULUS MECHANICS

The actomyosin cortex is a complex structure, capable of a wide range of mechanical behaviors [1]. We limited the scope of our model to describe the cortex under tension and its response to the microinjection and laser ablation experiments, which happen on a sub-second to second timescale. At this timescale, elastic response dominates.

A. Elastic energy

We model the BC as an elastic surface, the energy of which is given by

$$G = \int_{A_0} w \, \mathrm{d}A_0 - pV, \tag{1}$$

as used, e.g., in Ref. [2]. The second term is the is the contribution from the fluid inside the BC, where p is the pressure and V is the volume of the deformed body. The first term is the contribution from the elastic surface, which we calculate by integrating the surface energy density w over the undeformed surface, A_0. The surface energy density is given by

$$w = \frac{c}{2(1-\nu^2)} \left[\nu \operatorname{Tr} \boldsymbol{E}^2 + (1-\nu)(\operatorname{Tr} \boldsymbol{E})^2 \right], \tag{2}$$

where c is the surface elastic modulus. The Green-Lagrange strain tensor, $\boldsymbol{E}$, is defined to be

$$\boldsymbol{E} = \frac{\boldsymbol{F}^{\top}\boldsymbol{F} - \boldsymbol{I}}{2}, \tag{3}$$

where $\boldsymbol{F}$ is the deformation gradient and $\boldsymbol{I}$ is the identity, as also used in Ref. [3]; see Ref. [4] for a full treatment.

B. Implementation in Surface Evolver and definition of parameters

An energy equivalent to the one described above is implemented in Surface Evolver [5], in the named method `linear_elastic`. When provided with a set pressure, Surface Evolver minimizes the appropriate energy to minimize G.

The expanded shape of the BC is found by first establishing a preferred shape at which the energy of the system is set to zero, then minimizing the energy at increasing internal pressures to find a match with measured values (Section II). We construct the preferred shape for the BC by establishing a simple set of vertices, edges and faces with a custom script in Mathematica. We then read this simple geometry into Surface Evolver and manipulate and refine it to achieve the desired shape (which is not itself a minimum energy shape). The preferred shape of the BC is shown in Fig. 1(a). Once the preferred shape is established, shapes of the BC at higher pressures are found by setting a target volume and minimizing the energy of the system. The expanded shape for the final inferred parameters is shown both in main text Fig. 5 and reproduced in Fig. 1(b).

Each face in the Surface Evolver model has its own elastic modulus. Drawing from our understanding of the structure of the BC, we parameterize the mechanics of the BC by dividing it into three areas, as shown by the colors in Fig. 1. We create a ring around the BC to represent the mechanical contribution of the adherens junction (red). We give the bulkheads a different elastic modulus (green) from the rest of the cortex (grey).

II. PARAMETERIZATION OF THE MODEL FROM DEFLATION EXPERIMENTS

We examined images of BC in culture and found their unperturbed, expanded shape to be well described by a tube with rounded ends, inside of which a number of bulkheads are present. Bulkheads tended to be placed in an alternating fashion, and usually extended about one third to one half of the way into the lumen. Furthermore, BC tended to bulge out slightly between bulkheads. In these bulging areas, BC had approximately circular cross-section. We based the expanded shape of our model BC on these observations, and completed its parameterization by setting the length, width and number of bulkheads to the means of those measured for a set of BC (Supplementary Figure 3, Methods).

To define the preferred shape, we undertook experiments in which we intentionally deflated the BC by ablating the cortex at the apical membrane (main text Figure 4). After ablation, we observed that BC shrunk in volume—quickly initially, then more slowly until stopping within a few seconds. Stopping of the flow could indicate either that the plasma membrane has healed, stopping the flow while a pressure difference still exists between the lumen and cytoplasm, or that the pressure has equalized. Since this timescale is fast compared to the turnover of actin [1], even if the membrane recovered it is unlikely that it could hold much of a pressure difference. Furthermore, we tested whether pressure was equalized between the lumen and cytoplasm at the end of the initial deflation period by ablating the cortex once, waiting for the BC to deflate, then ablating the cortex again. Little to no size change was observed for the lumen after the second ablations, indicating that the pressure is equalized between the two compartments. Therefore, we equate the shape after deflation with the preferred shape of the BC. We also note that bulges present before ablation were absent after deflation.

Width and length were measured after deflation for the same set of BC as before ablation. BC width changed significantly, but BC height did not change measurably after ablation. Therefore we set the height of the preferred shape of the BC equal to

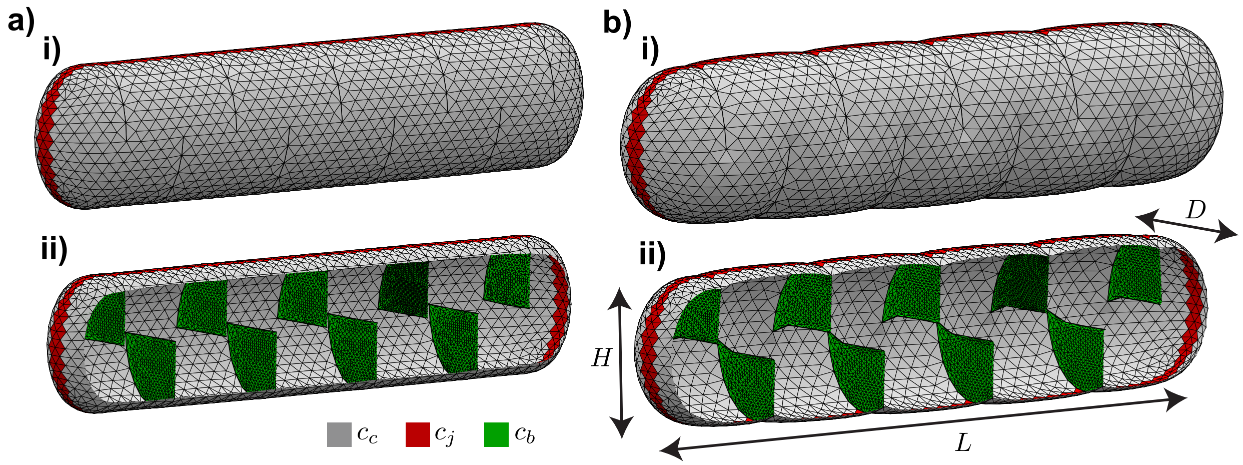

FIG. 1. BC shapes. (a) Rest shape, view from outside (i) and into the inside (ii). (b) Inflated shape, view from outside (i) and into the inside (ii). Grey areas represent the cortex surrounding the BC and have elastic modulus c_c. Red areas represent the mechanical contribution of the junctions, and have elastic modulus c_j. Green areas are bulkheads with elastic modulus c_b.

Parameter	Description	Value	Determination
c_b/c_c	Bulkhead elastic modulus	1.2	Parameter inference
c_j/c_c	Junction elastic modulus	3.5 c_b/c_c	deflation experiments
ν	Poisson's Ratio	0.5	Assumed
t	Cortex thickness	200 nm	Electron microscopy from Ref. [6]
p/C_c	Pressure of fluid inside the BC	0.2	Adjusted to fit expanded shape
D_*	Diameter of expanded BC shape	3.6 µm	measured before deflation
L_*	Length of expanded BC shape	17.6 µm	measured before deflation
N_b	Number of bulkheads per length	0.54 μm^{-1}	measured
W_p	Preferred width of BC shape	2.8 µm	measured after deflation
H_p	Preferred height of BC shape	3.6 µm	equal to diameter before deflation
L_p	Preferred length of BC shape	16.8 µm	measured after deflation
D_{bleb}/D_*	Diameter increase before blebbing	1.2	Measured from microinjection

TABLE I. List of model parameters and their values. Mechanical parameters are normalized to the cortex elastic modulus, c_c (units Pa µm), or in the case of the pressure, the cortex Young's modulus $C_c = c_c/t$ (units Pa).

the width of the expanded shape, as BC are generally circular in cross section before deflation. Neither the number nor the depth of the bulkheads changed during deflation. Because BC rarely had either bulges or indented regions along the sides at bulkhead locations after ablation, we set the preferred width of the bulkheads to be equal to the preferred width of the BC.

With the preferred shapes defined, we moved on to examine the mechanical parameters of the model - the three elastic moduli we defined, as well as the pressure. In this set of 4 parameters, only 3 are identifiable. Were each multiplied by a common factor, the resulting shape would be the same. We choose to normalize by the modulus of the cortex. We note that unlike Young's modulus in a full 3D model, the surface elastic modulus does not have the same units as pressure. Therefore we relate the cortex modulus to the pressure by multiplying it by the approximate thickness of BC cortices as revealed by electron microscopy by Belicova *et al.* [6].

The expanded shape of the model BC is found for a particular set of mechanical parameters in the following way. The preferred shape is fully defined from measurements. We inflate this shape in Surface Evolver to find a critical volume V_* at which $D = D_*$. This volume varies slightly depending on the values of the moduli. We found that for a variety of bulkhead moduli we were able to match the measured 5% change in length at V_* by setting the junction modulus to 3.5 times that of the bulkheads.

III. PARAMETER INFERENCE ON BULKHEAD MODULUS

All parameters but the bulkhead modulus are set by measuring the shapes of BC before and after deflation. Further quantification of shapes is limited by image quality and resolution, so we use the data on the expansion ratio after bulkhead ablation to infer it.

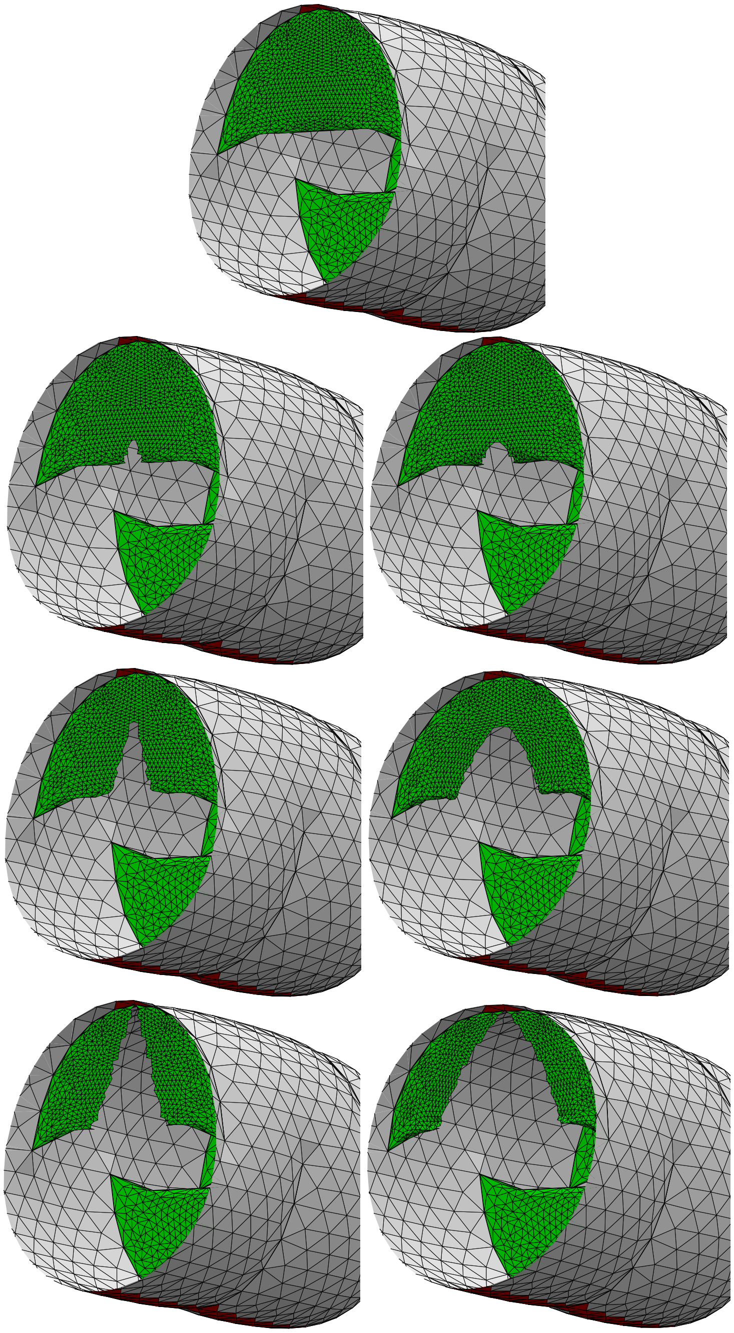

FIG. 2. Visualization of bulkhead ablations at different depths. Rows, from top to bottom: bulkhead before ablation, 0.38 µm ablation depth, 1.07 µm ablation depth, 1.77 µm ablation depth. Left: ablated shape before energy minimization. Right: ablated shape after energy minimization.

A. Ablation of bulkheads in the model

To simulate expansion ratios, bulkhead ablations were implemented in the Surface Evolver model. This was accomplished by choosing a set of triangles in one of the bulkheads and setting their elastic modulus to a value 1000 times smaller than that of the cortex. We picked ablation areas to correspond roughly to the shape of a confocal volume, with a depth 1.5 times the width (as one would expect for ablation by half a confocal volume with a z-extent thrice that of the x, y-extent. Example ablations at three depths are shown in Fig. 2.

Expansion ratios were measured by examining the set of vertices at which the bulkhead connects to the surrounding cortex, as shown in Fig. 3. The difference between the pre- and post-ablation bulkhead profiles was evaluated at ridge of the bulkhead, mimicking measurement at the plane at which the microscope was focused during ablation experiments.

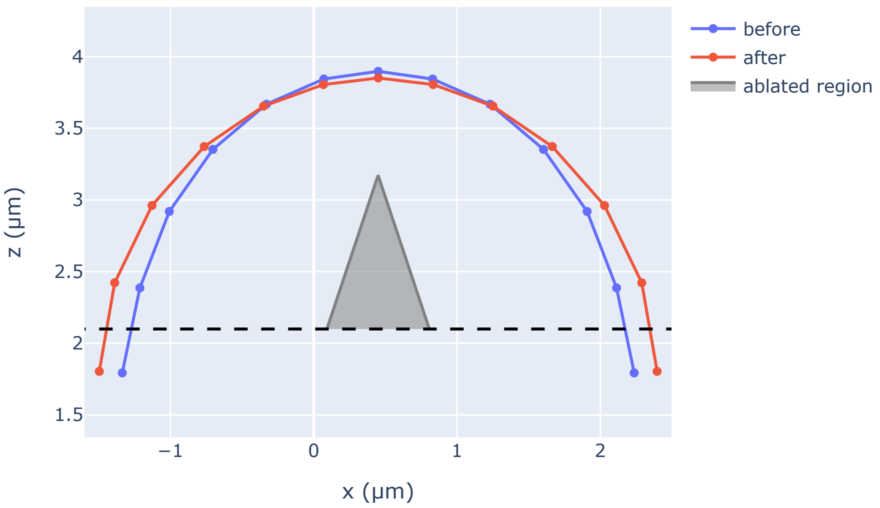

FIG. 3. Visualization of expansion ratio measurement. When simulated bulkheads are ablated, the BC expands at the bulkhead location. The vertices defining the intersection of the bulkhead with the rest of the BC are shown before and after ablation. The degree of expansion depends on the z coordinate at which the the widths are measured. We measure expansion at the ridge of the bulkhead, as shown by the black dashed line, to mimic the placement of the image plane in the ablation experiments.

B. Existence of a maximum expansion ratio

As shown in Fig. 4, we find that for a given ablation depth, there exists a maximum expansion ratio. It is not immediately clear why this should be the case, but the existence of this maximum can be understood as follows. If there were no bulkheads, the expanded shape of the BC would be a smooth tube. The width of a bulkhead in this expanded shape is determined by how much the bulkhead pulls the cortex in from this no-bulkhead shape. Furthermore, the expansion ratio is determined by how far the surrounding cortex is able to pull back toward this no-bulkhead shape once the ablation is done. If the bulkheads were to have a modulus of zero, there would have no ability to pull the cortex in, and therefore no expansion would happen on ablation. Next, we consider a bulkhead with very large modulus. In the expanded shape, the bulkhead stays very close to its rest shape. In fact, with high enough modulus the bulkhead would stay the same shape after ablation as well, as the remaining material will not deform even without the support of the material which was removed. Therefore, we expect the expansion ratio to be zero in both the limit as bulkhead modulus goes to zero and to infinity, meaning there must be a peak in expansion ratio somewhere in between. With our simulations, we find that peak occurs at a bulkhead modulus near 10, as shown in Fig. 4.

C. Inference on the range of consistent bulkhead moduli

Observing a given expansion ratio is possible only beyond a minimum cut depth. Furthermore, we find cuts deeper than the extent of the bulkheads to be unlikely in experiments due to limited deflation of the lumen after ablation. This establishes a maximum cut depth. These limits on the cut depth together impose limits on the bulkhead moduli consistent with each expansion ratio measurement. We find the lower limit by evaluating an interpolation of the simulation data at the maximum ablation depth (Fig. 4, black line). We find the upper limit by evaluating a curve fit through the maximum values of ablation depth as a function of cortex modulus (Fig. 4, dashed line).

Each experimentally measured expansion ratio yields a range of consistent moduli. There is a range of moduli which are consistent with all data (Fig. 4, gray). We note that we excluded one data point with an expansion ratio significantly higher than the others from this analysis. Our model has a fixed geometry, while data points come from a variety of different actual geometries. The data point removed was found to come from a BC with geometry much different from the mean geometry represented by our model. Removing any other data point alters the outcome of the inference by at most 10%.

D. Finding the most likely bulkhead modulus

Our simulation results reveal ranges of bulkhead moduli consistent with any measured expansion ratio. By further assuming a probability distribution of ablation depths within this range, we infer a probability distribution of bulkhead moduli. We then show the most likely modulus we infer is insensitive to the choice of ablation depth distribution within reasonable limits.

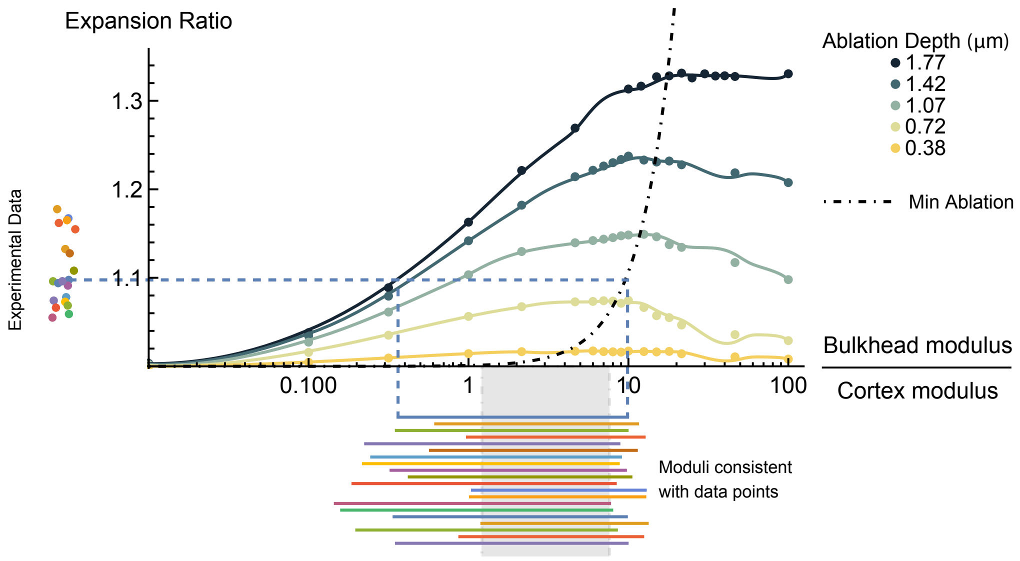

FIG. 4. Expansion ratios resulting from simulated ablations and ranges of moduli consistent with experimental data. Simulated expansion ratios (dots) are shown as a function of bulkhead modulus for a variety of ablation depths, along with interpolating curves. Also shown is the expansion ratio as a function of the modulus consistent with the minimum ablation depth at which that expansion ratio can be observed (black dashed curve). For each experimentally measured expansion ratio (dots to left of axis) the moduli between the maximum ablation depth (solid black curve) and the minimum ablation depth (dashed black curve) are consistent with that data point. Blue dashed lines indicate the matching points for an example measured expansion ratio. Solid colored lines below the axes represent the ranges of moduli consistent with each measured data point. The range highlighted in grey is consistent with all measured expansion ratios.

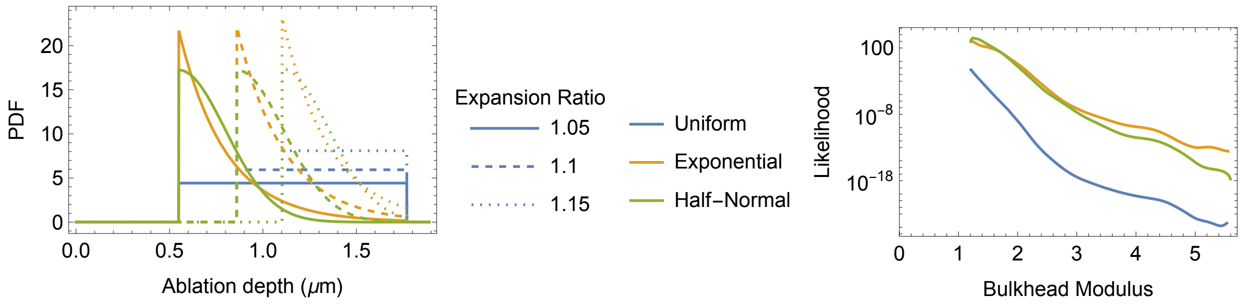

FIG. 5. Inference on likelihood of bulkhead modulus for different assumed ablation depth distributions. Left: Probability density functions of assumed uniform, exponential and half-normal distributions. Each is a function of the measured expansion ratio and three example values are shown solid, dashed and dotted. Standard deviations of exponential and half-normal distributions are set to 250 nm to reflect accuracy of bulkhead targeting in the microscope. Right: Likelihood of the value of the bulkhead modulus for each of the three assumed ablation depth distributions.

1. *Uniform distribution of cut depths*

We begin by assuming a uniform distribution of cut depths between an ablation depth of 0 and the depth of the bulkhead. We truncate this distribution at the minimum depth consistent with a measured expansion ratio to define an ablation depth distribution $p(d|r_i)$ (Fig. 5, blue curves). Then, we seek the distribution of bulkhead moduli resulting from this distribution of ablation depths. We accomplish this numerically in the following manner. From the Surface Evolver simulation output, we have a sampling of expansion ratios as a function of bulkhead modulus and ablation depth. We construct a function $r(c, d)$ by interpolating these points. Then, for each measured expansion ratio r_i we take 1000 samples from $p(d|r_i)$. For each sample d_j, we solve $r(c_j, d_j) = r_i$ for c_j. Then we construct the distribution $p(c_i)$ by fitting a Bezier curve to the empirical cumulative distribution function of the set of c_js and taking its derivative. To find the likelihood of c, we multiply together all distributions $p(c_i)$. We find that the lowest modulus consistent with all data points is the most likely (Fig. 5).

2. *Other cut depth distributions*

For a given expansion ratio, the shallower the cut one presumes is responsible, the higher the bulkhead modulus one infers (and vice versa). It is therefore possible that if the experimental distribution of cut depths were skewed toward shallow cuts, the most likely modulus would be different from the one found for a uniform distribution. We repeat the process above with exponential and half normal distributions for d. We find that if we allow the standard deviations of the distributions to go arbitrarily low, we are indeed able to find peaks in likelihood above the minimum. To estimate the tightest distribution that could have been achieved in experiments, we considered how accurately the ablation laser could be aligned with the bulkhead. Because bulkhead z positions were found by eye and hand adjustment of the microscope focus, we estimate $\sim$250 nm maximum accuracy. When we set the standard deviations of the trial distributions to reflect this accuracy, we find that the maximum likelihoods remain at the lowest consistent bulkhead modulus as shown in Fig. 5.

3. *Discussion of inferred value*

The value we infer for the modulus of the bulkheads is 1.2 times the cortex modulus. This is consistent with our observations that the cortex surrounding the BC extends into the bulkheads. If one assumes the cortex in the bulkheads is similar to the surrounding cortex, then one might guess that the bulkhead modulus is between one and two times that of the surrounding cortex, as cortex from either side meets and merges in bulkheads.

IV. DISCUSSION OF BC SHAPES AND PRESSURES WITH AND WITHOUT BULKHEADS

The outcome of our parameter inference is a fully parameterized model for BC mechanics, which is based on the BC having a circular cross-section when expanded as observed in experiments. The result of changing pressure in this parameterized model is a change in shape - if pressure is decreased, the shape of the BC goes toward the narrower elastically preferred shape (Figure 1). If the pressure is increased, the BC expands beyond a circular cross-section, eventually creating muffin-like bulges around the less-expandable junction ring.

We compare BC with and without bulkheads and find that BC with bulkheads have circular cross-section at a higher pressure than BC without bulkheads have circular cross-section. In main text figure 5, we show expansion of the BC as an increase of diameter, as this is the observable variable in our microinjection experiments. We note that one could also think of this expansion in terms of stretch in the cortex - the larger the diameter we observe, the more the cortex must have stretched. Because the amount of stretch is proportional to tension and the amount of stretch is similar when the BC with and without bulkheads have circular cross-section, our results also show that BC with bulkheads hold double the pressure at the same cortical tension as a BC without bulkheads.

[1] G. Salbreux, G. Charras, and E. Paluch, Actin cortex mechanics and cellular morphogenesis, Trends Cell Biol. **22**, 536 (2012).

[2] S. Knoche and J. Kierfeld, Buckling of spherical capsules, Phys. Rev. E **84**, 046608 (2011).

[3] A. L. Gregory, J. Lasenby, and A. Agarwal, The elastic theory of shells using geometric algebra, Roy. Soc. Open Sci. **4**, 170065 (2017).

[4] A. Libai and J. Simmonds, *The Nonlinear Theory of Elastic Shells*, 2nd ed. (Cambridge University Press, Cambrige, 1998).

[5] K. A. Brakke, The Surface Evolver, Exp. Math. **1**, 141 (1992).

[6] L. Belicova, U. Repnik, J. Delpierre, E. Gralinska, S. Seifert, J. I. Valenzuela, H. A. Morales-Navarrete, C. Franke, H. Räägel, E. Shcherbinina, T. Prikazchikova, V. Koteliansky, M. Vingron, Y. L. Kalaidzidis, T. Zatsepin, and M. Zerial, Anisotropic expansion of hepatocyte lumina enforced by apical bulkheads, J. Cell Biol. **220**, e202103003 (2021).
